# 9-Gene Signature Correlated With CD8^+^ T Cell Infiltration Activated by IFN-γ: A Biomarker of Immune Checkpoint Therapy Response in Melanoma

**DOI:** 10.3389/fimmu.2021.622563

**Published:** 2021-06-17

**Authors:** Kexin Yan, Yuxiu Lu, Zhangyong Yan, Yutao Wang

**Affiliations:** ^1^ Department of Dermatology, China Medical University, The First Hospital of China Medical University, Shenyang, China; ^2^ Department of Pharmacy, Fuzhou No. 1 Hospital Affiliated With Fujian Medical University, Fuzhou, China; ^3^ Department of Stomatology, Fuzhou No. 1 Hospital Affiliated with Fujian Medical University, Fuzhou, China; ^4^ Department of Urology, China Medical University, The First Hospital of China Medical University, Shenyang, China

**Keywords:** CD8^+^ T cells, weighted gene co-expression network analysis (WGCNA), melanoma, immune microenvironment, immunotherapy

## Abstract

**Purpose:**

To identify CD8^+^ T cell-related factors and the co-expression network in melanoma and illustrate the interactions among CD8^+^ T cell-related genes in the melanoma tumor microenvironment.

**Method:**

We obtained melanoma and paracancerous tissue mRNA matrices from TCGA-SKCM and GSE65904. The CIBERSORT algorithm was used to assess CD8^+^ T cell proportions, and the “estimate” package was used to assess melanoma tumor microenvironment purity. Weighted gene co-expression network analysis was used to identify the most related co-expression modules in TCGA-SKCM and GSE65904. Subsequently, a co-expression network was built based on the joint results in the two cohorts. Subsequently, we identified the core genes of the two most relevant modules of CD8^+^T lymphocytes according to the module correlation, and constructed the signature using ssGSEA. Later, we compared the signature with the existing classical pathways and gene sets, and confirmed the important prognostic significance of the signature in this paper.

**Results:**

Nine co-expressed genes were identified as CD8^+^ T cell-related genes enriched in the cellular response to interferon−gamma process and antigen processing and presentation of peptide antigen. In the low expression level group, inflammation and immune responses were weaker. Single-cell sequencing and immunohistochemistry indicated that these nine genes were highly expressed in CD8^+^ T cells group.

**Conclusion:**

We identified nine-gene signature, and the signature is considered as the biomarker for T lymphocyte response and clinical response to immune checkpoint inhibitors for melanoma

## Introduction

Melanoma is one of the deadliest forms of skin cancer. In addition to external causes such as ultraviolet radiation, familial genetic factors cannot be ignored. Patients with melanoma diagnosed early and treated surgically had higher survival rates, and those with advanced metastatic melanoma had lower survival rates (1). In recent years, melanoma treatment has included conventional chemotherapy and radiation therapy and therapies targeting B-raf proto-oncogene, serine/threonine kinase (BRAF), and MAP kinase-ERK kinase. The emergence of immune checkpoint inhibitors has extended survival from metastatic melanoma. Ipilimumab, an anti-cytotoxic T-lymphocyte-associated protein 4 (CTLA-4) antibody, and the programmed cell death protein (PD-1) antibodies nivolumab and pembrolizumab are now widely used for immunotherapy (2).

PD-1, an immune checkpoint protein on T cells, combines with programmed cell death ligand 1 (PD- L1) to inhibit the activity of lymphocytes in the peripheral circulation, thereby inhibiting inflammation and immune response. In the tumor microenvironment, PD-1 has a promoting effect on the immune escape mechanism. Unlike CTLA-4, PD-1 can be induced on the surface of T cells and activated B lymphocytes and NK cells. This fact suggests that blocking PD-1 can rejuvenate disabled immune cells (3). ICI targeting cytotoxic T lymphocyte antigens, or PD-1 receptors, activates T cells and other immune cells, allowing the immune system to attack melanoma (4).

Despite the great success of immunotherapy, there remain many patients unresponsive to or resistant to PD-1 blockers. The reasons for this resistance include lack of CD8^+^ T cells associated with endogenous oncogenic activation of the WNT-β-catenin pathway in melanoma cells (5), lack of PD-1 receptors, mutation and antigen presentation of tumor antigen, dynamic changes of immune microenvironment, genetic mutations, and epigenetic changes in essential tumor proteins (6).

CD4^+^ and CD8^+^ T cells participate in cellular immunity. The former, also known as helper T lymphocytes, assist humoral and cellular immunity. The latter, also known as cytotoxic cells, kill target cells (7). Most tumor cells express antigens that mediate the recognition of CD8^+^ T cells. Infiltration of CD8^+^ T cells and high expression of the immune system have important clinical significance for immunotherapy of cancer suppression (8). Therefore, in the present study, we explored the mechanism related to CD8^+^ T cell infiltration based on the WGCNA method to identify the gene signature.

CIBERSORT packages (9) and weighted gene co-expression network analysis (WGCNA) were used to determine the co-expression factors and related biological functions associated with CD8^+^ T cell infiltration. The results were validated in two datasets (10).

We identified nine co-expressed genes correlated with CD8^+^ T cell infiltration in melanoma, possibly involved with the biological process of IFN-γ. Next, we measured the correlations between the 9-gene signature expression with angiogenesis, wound healing, and immune responses. Finally, we determine that these factors inhibited tumor proliferation and differentiation, thereby confirming our hypothesis.

The proposed 9-gene signature contains genes related to T cell activation, cytotoxicity, and the regulation of IFN-γ downstream, which may indicate that this signature can affect CD8^+^ T cells through IFN-γ activation in melanoma. In addition, by comparing several classical scores previously proposed, we found that 9-gene signature not only has a good predictive ability for the prognosis and survival of melanoma patients, but also can be used as one of the biomarkers to evaluate the response to immunotherapy.

## Materials and Methods

### Data Source

SKCM-FPKM data were downloaded from The Cancer Genome Atlas (TCGA) (http://cancergenome.nih.gov/), containing 470 melanoma cancer tissue samples. GSE65904 (11) was downloaded from the GEO (http://www.ncbi.nlm.nih.gov/geo/) database whose platform is GPL10558. GSE65904 contained 214 melanoma cancer samples. GSE65904 contained 214 melanoma cancer samples. GSE78220 (12), GSE72056 (13), GSE91061 (14) and GSE93157 (15) were downloaded from the GEO database and used for subsequent validation.

### CD8^+^ T Cell Proportions and Tumor Purity

CIBERSORT is an algorithm for analyzing the proportion of cells in tumor tissues (9). LM22 represents the genetic markers of 22 immune cell subtypes; it was downloaded from the CIBERSORT website portal (https://cibersort.stanford.edu/). Based on this method, we analyzed the proportion of CD8^+^ T cells, and a P value less than 0.05 was considered statistically significant. Using expression data to estimate stromal cells and immune cells in tumor tissues (ESTIMATE) (16), we evaluated the tumor purity of melanoma based on the ESTIMATE algorithm.

### Weighted Gene Co-Expression Network Analysis

We know that the gene expression patterns involved in the same pathway or biological process are similar (17). WGCNA constructs a co-expression network by converting co-expression correlation into connection weight or topological overlap value. Based on this background, we used WGCNA to construct a co-expressed gene network related to the relative content of CD8^+^ T cells (10). We normalized the TCGA-SKCM data with Log2(exp+1). We set the soft threshold to 5, R square = 0.89 in TCGA-SKCM, R square = 0.88 in GSE65904, and the number of genes in the smallest module to 30.

### Protein-Protein Network and Functional Enrichment

After identifying the modules most related to CD8^+^ T cells, we used Pearson correlation coefficients and module correlations to screen CD8^+^ T cell-related genes. We performed an intersection analysis in the two cohorts based on module correlations greater than 0.7 and CD8^+^ T cells correlations greater than 0.4. Cytoscape software was used to generate the protein-protein interaction network of the intersection factor. Kyoto Encyclopedia of Genes and Genomes (https://www.genome.jp/kegg/) (18) and Gene Ontology (GO) (http://geneontology.org/) (19) were used to determine the biological functions of these intersection factors. These methods were implemented in the database DAVID, v6.8 for annotation, visualization, and integrated discovery (20).

### Immune Microenvironment Correlation Analysis

In GSE65904 and TCGA-SKCM, we investigated the correlation between CD8^+^ T cell-related genes and immune responses in melanoma, then drew the heat map. We selected several classic immune-related metagene sets, including major histocompatibility complex class I (MHC-1), major histocompatibility complex class II (MHC II), lymphocyte specific kinase (LCK), hematopoietic cell kinase (MCK), immunoglobulin G (IgG), signal transduction and activation transcription 1 (STAT1) and interferon (21).

### Gene Set Enrichment Analysis

Gene set enrichment analysis (GSEA) can determine the meaning and differences between two biological states using a predefined data set (22). We divided the gene matrix in TCGA into high expression and low expression groups according to the median of the proportion of CD8^+^ T cells. We then determined the mechanism of CD8^+^ T cell infiltration-related co-expressed genes.

### Timer

The Tumor Immune Estimation Resource (TIMER; https://cistrome.shinyapps.io/timer/) (23) was used to identify the correlations between CD8^+^ T cells and other types cancer. Pearson correlation coefficients higher than 0.4 were considered significant.

### Single-Cell Sequencing

Single-cell cohort data sets GSE72056 were analyzed to determine the correlation between CD8^+^ T cell and co-expression genes. GSE72056 contains 4645 single-cell sequencing samples, and the platform is GPL18573 Illumina NextSeq 500 (Homo sapiens). We used the “seurat” package to filter the sequencing data, batch calibration, and data standardization. Then, we used the “t-SNE” package to perform cell cluster analysis. Finally, we used “SingleR” to perform cell subpopulation annotation identification. By measuring the expression level of each cell, we demonstrated correlations between CD8^+^ T cells and co-expressed genes.

### ssGSEA

Based on the above methods, we screened a gene signature, then ssGSEA was used to score the gene set. Based on these scores, we explored the prognostic value of gene signature in different melanoma cohorts and in different types of cancers. Meanwhile, in TCGA-SKCM, the prognostic significance of ssGSEA score of the gene signature was compared with the more classical gene sets at present. For instance, CYTOLYTIC score by Rooney et al (24), hallmark angiogenesis gene set and hallmark interferon gamma response gene set from the molecular signature database (25), immune response-related gene sets, CD4^+^T cell score and CD8^+^T cell score by CIBERSORT.

### Immunohistochemical Verification

Archives were registered from recruited melanoma patients, and resection and biopsies of freshly collected melanoma and para-tumor tissue were performed. All participants provided written informed consent. The Ethics Committee of Fuzhou No. 1 Hospital Affiliated with Fujian Medical University approved the study. The extracted human tissues were fixed with 4% formaldehyde buffer. Deparaffinized specimens were then sectioned into 4-µm sections. Tissue sections were incubated at 60°C for 2 h before dewaxing, autoclaved at 115°C for 3 min for antigen retrieval in a citric acid buffer (pH 6.0), and quenched for endogenous peroxidase activity with 0.3% H_2_O_2_ solution for 15 min. Then, the sections were blocked with normal goat serum for 45 min and incubated with the specific primary antibody against PSMB10 (dilution 1:200) overnight at 4°C. Subsequently, the sections were treated with goat anti-mouse secondary antibody for 30 min at room temperature. Protein expression was visualized using 3,3′-diaminobenzidine. Images were captured using a Nikon Eclipse 80i microscope (Nikon Corporation). The immunohistochemical integral optical density (IOD) was analyzed by ImageJ. Fifteen pairs of IOD with PSMB10 expression levels in melanoma tumors and paracancerous tissues were submitted to GraphPad Prism. The IOD sum and area of each photo were measured, and the average optical density between groups was compared.

### Statistical Analysis

Statistical analysis was carried out using GraphPad Prism 8 and R 3.6.3 (https://www.r-project.org/). The subgroups were divided based on the median value. Kaplan–Meier survival analysis was used to generate overall survival curves, and the log-rank test was used to calculate the significance. The nine gene score was evaluated using single-sample gene set enrichment analysis (ssGSEA). The “survival”, “ggplot2”, “corrplot”, “pheatmap”, “GSVA” and “limma” packages were built using R version 3.6.3. Differences with P < 0.05 were considered significant.

## Results

### Identification of CD8^+^ T Cell Infiltration Modules and Genes

**Figure 1:**
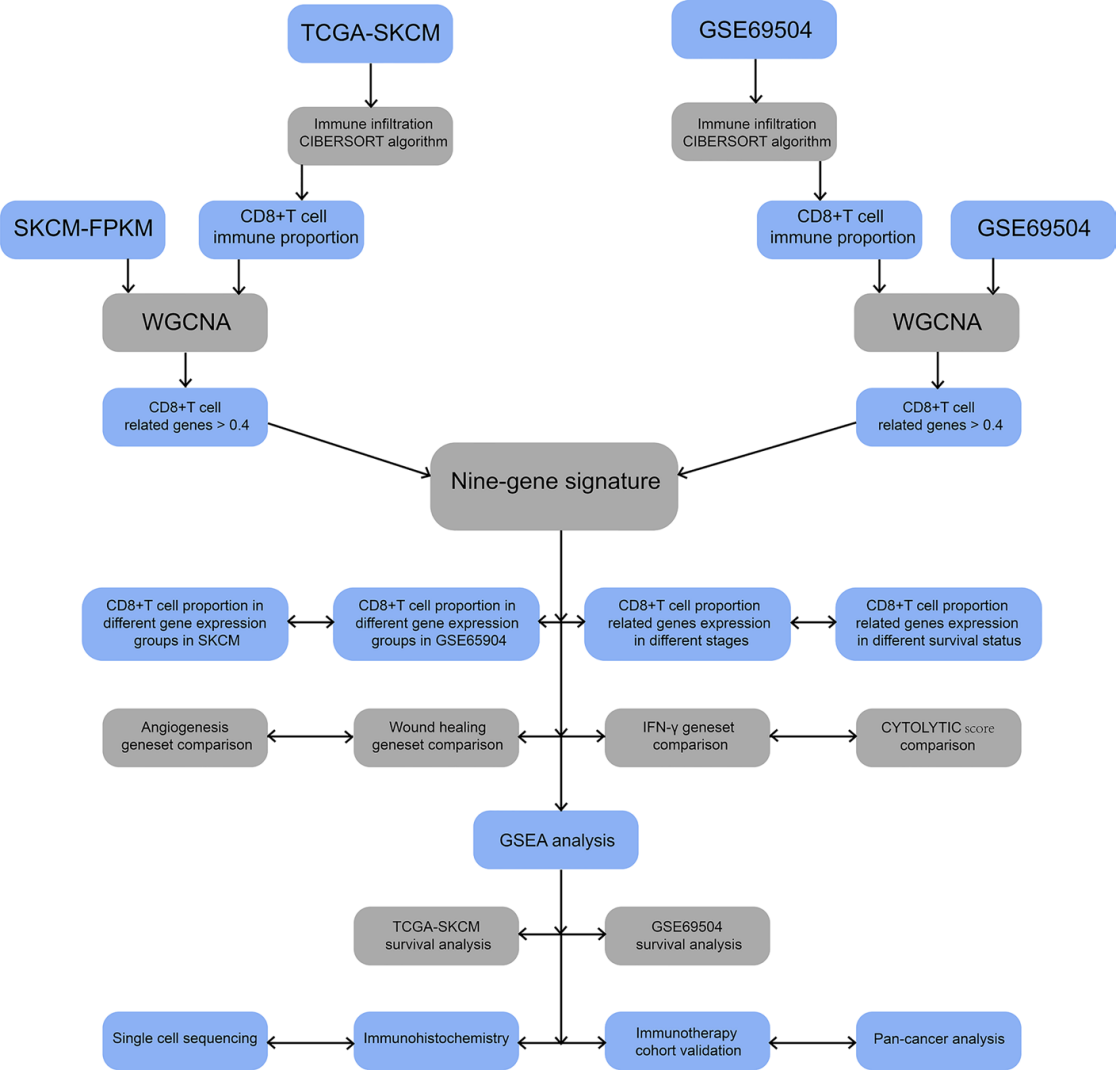
Flowchart for identification of co-expressed genes promoting CD8^+^ T cells infiltration. SKCM-FPKM contains 470 melanoma cancer tissue samples. GSE65904 contains 214 melanoma samples. CIBERSORT algorithm was applied to calculate the infiltration of CD8^+^ T cells in melanoma tissue samples from two datasets. WGCNA was used to generate a co-expressed gene network, and the two datasets were intermingled to obtain nine essential genes. The main pathways of critical gene enrichment were obtained in GSEA analysis. Survival analysis was used to determine the influence of critical genes on the outcome. Single-cell sequencing and immunohistochemical experiments confirmed that the expression of these genes was high in CD8^+^ T cells. The comparison between 9-gene signature with angiogenesis geneset, wound healing geneset, immune response geneset and IFN-γ genset were analyzed. Follow-up cohorts with immunotherapy were also used to validate the role of critical genes in immunotherapy. The 9-gene signature that related to CD8^+^ T cell infiltration in other cancers were verified.

**Figure 2:**
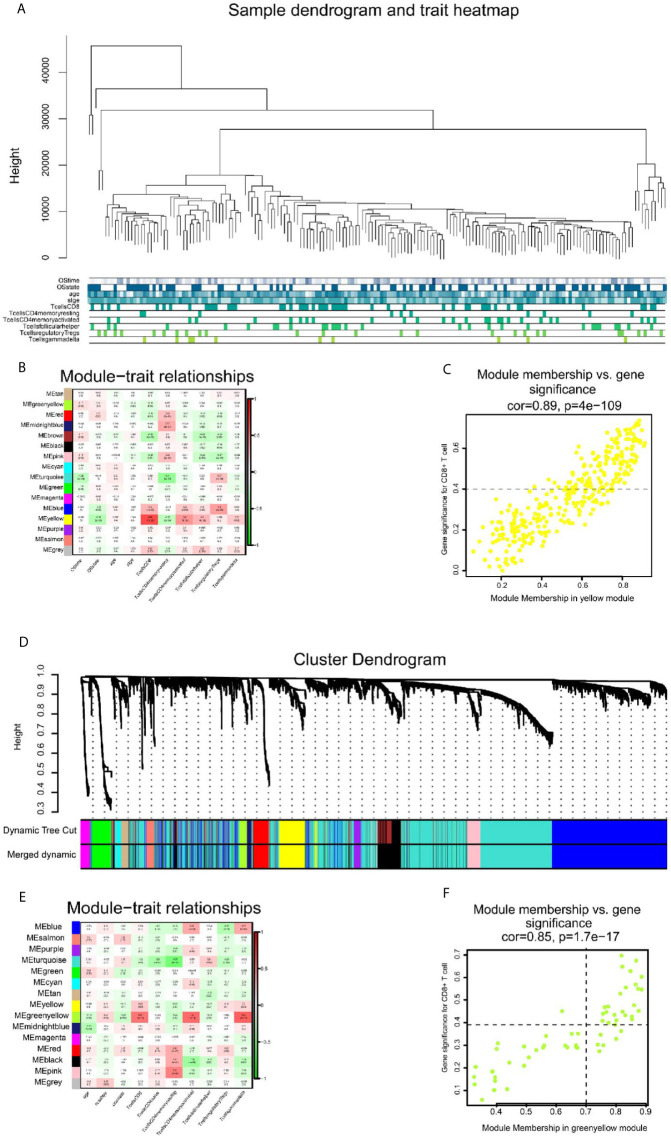
The result of WGCNA analysis in SKCM-FPKM and GSE65904. **(A)** 470 samples were clustered according to cut Height = 46000, and a total of 177 samples were included. Tree and trait graphs of 177 samples are presented. **(B)** Correlation coefficients between different phenotypes and co-expression modules were shown. The yellow module had the highest correlation with CD8 cells (R = 0.69, P = 1e-26). **(C)** Gene significance for CD8^+^ T cell = 0.4, module membership in yellow module = 0.7 (cor = 0.89, P = 4e−109). **(D)** Hierarchical cluster trees are constructed by dynamic hybrid cutting method, in which each leaf represents a gene, and each branch represents a co-expression module. A total of 15 co-expression modules were produced. **(E)** Correlation coefficients between different phenotypes and co-expression modules were shown. The yellow-green modules had the highest correlation with CD8^+^ T cells (R² = 0.57, P = 4e-11) **(F)** Gene significance for CD8^+^ T cell = 0.4, module membership in green-yellow module=0.7. (cor = 0.85, p = 1.7e−17).

The flow chart of this study is illustrated in **Figure 1**. We obtained 202 TCGA-SKCM and 143 GSE65904 samples with complete clinical information and proportion of immune cell infiltration assessment after selection by P < 0.05. The ratio of 22 immune cells is shown in **Supplementary Figures 1A, C** (refer to **Supplementary Table 1**for the original data). The tumor purity heat map is shown in **Supplementary Figures 1B, D**. We performed cluster analyses in TCGA-SKCM and GSE65904. The cluster heat map of 178 samples in TCGA-SKCM is shown in **Figure 2A**, and the correlation coefficients between each phenotype and co-expression module are shown in **Figure 2B**. We found that the yellow module had the strongest correlation with CD8^+^ T cells in the TCGA-SKCM cohort (Cor = 0.69; P= 1e-^26^). Based on these findings, we supplemented the heat map of the correlation between the yellow module and CD8^+^ T cells and the yellow module (**Figure 2C**) (the module correlation coefficient was greater than 0.7; the CD8^+^ T cell correlation coefficient was greater than 0.4).

**Figure 3:**
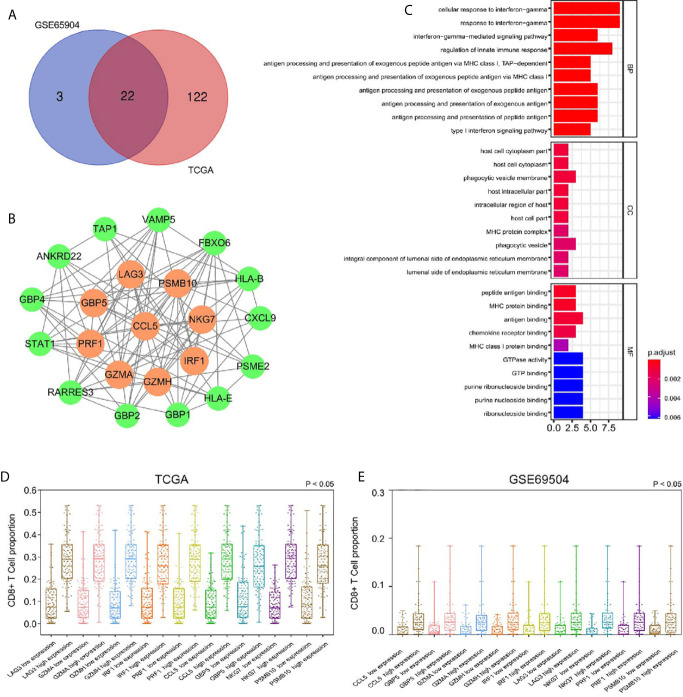
**(A)** Identification of 22 factors in the intersection of the two modules. **(B)** The protein-protein network and the nine factors that were most closely connected. The orange circles represent nine genes with P-values less than 0.0001 in the survival analysis. The green circles represent other genes with P-values less than 0.05 but not less than 0.001 in the survival analysis. **(C)** According to GO analysis, in the BP, essential genes are enriched in the IFN-γ−mediated signaling pathway and antigen processing and presentation of exogenous antigen pathway. **(D)** The high expression group had a higher proportion of CD8^+^ T cells infiltration, suggesting that these genes related to CD8^+^ T cells infiltration in SKCM-FPKM. **(E)** The high expression group had a higher proportion of CD8^+^ T cells infiltration, suggesting that these genes related to CD8^+^ T cells infiltration in GSE65904.

We used the dynamic hybrid cutting method to build a hierarchical clustering tree. Each leaf on the tree represents a gene, and each branch represents a co-expression module in GSE65904 (**Figure 2D**). A total of 15 co-expression models were generated, and the correlation coefficients between each phenotype and co-expression module were calculated (**Figure 2E**). The heat map of the correlation between the green-yellow module and CD8^+^ T cells (**Figure 2F**) (the module correlation coefficient was greater than 0.7; the CD8^+^ T cell correlation coefficient was greater than 0.4). The genetic significance of the first 20 CD8^+^ T cells-related genes in the yellow module of TCGA-SKCM is shown in **Supplementary Table 2**. The genetic significance of the first 20 CD8^+^ T cells-related genes in the green-yellow module of GSE65904 is shown in **Supplementary Table 3**. Next, we screened intersection factors between the two modules based on the module correlation greater than 0.7 and the CD8 correlation greater than 0.4. Twenty-two factors were obtained (**Figure 3A**). Subsequently, in the 22 genes we identified core genes that could represent TCGA-SKCM and GSE65904 co-expressed modules. These core genes are defined as the correlation of module greater than 0.8 in both TCGA-SKCM and GSE65904 cohorts (**Table 1**). Their protein-protein interaction network is shown in **Figure 3B**. The nine core genes were marked as orange circles. We conducted functional enrichment of these intersection factors and found that the interferon response was the most significant, as shown in **Figure 3C**.

**Table 1:** TCGA-SKCM and GSE65904 intersection genes with CD8+T cells correlation.

id	TCGA-SKCM		GSE65904	
	CD8+T cell correlation	yellow module correlation	CD8+T cell correlation	green-yellow module correlation
CCL5	0.7150	0.8794	0.7056	0.8171
IRF1	0.7038	0.8574	0.6116	0.8846
GZMA	0.6677	0.8526	0.6370	0.8473
GBP5	0.6399	0.8512	0.5557	0.8847
NKG7	0.6740	0.8396	0.6837	0.8661
LAG3	0.6934	0.8379	0.5585	0.8724
PRF1	0.6907	0.8179	0.5374	0.8131
PSMB10	0.6892	0.8135	0.5645	0.8315
GZMH	0.6634	0.8057	0.6572	0.8200
CXCL9	0.5661	0.7760	0.4578	0.8205
ANKRD22	0.6262	0.7749	0.4231	0.8519
PSME2	0.6480	0.7700	0.4422	0.8299
HLA-E	0.6312	0.7596	0.4081	0.7733
RARRES3	0.6447	0.7460	0.4358	0.8517
STAT1	0.6045	0.7460	0.4562	0.7666
TAP1	0.6763	0.7230	0.4611	0.8741
HLA-B	0.5427	0.7200	0.4194	0.8001
GBP4	0.5620	0.7171	0.4439	0.7933
GBP2	0.4917	0.6925	0.4482	0.7552
GBP1	0.5158	0.6861	0.4861	0.8593
VAMP5	0.5186	0.6097	0.4774	0.7607
FBXO6	0.5498	0.5957	0.4174	0.7534

To calculate the correlations between these and CD8^+^ T cell infiltration proportions, subgroups were created according to the median of nine gene expression values in the TCGA-SKCM (**Figure 3D**) and GSE65904 (**Figure 3E**) cohorts. We found higher infiltration proportions in high expression groups (P < 0.05), suggesting that these genes are related to CD8^+^ T cell infiltration.

### Immune Microenvironment Analysis

**Figure 4:**
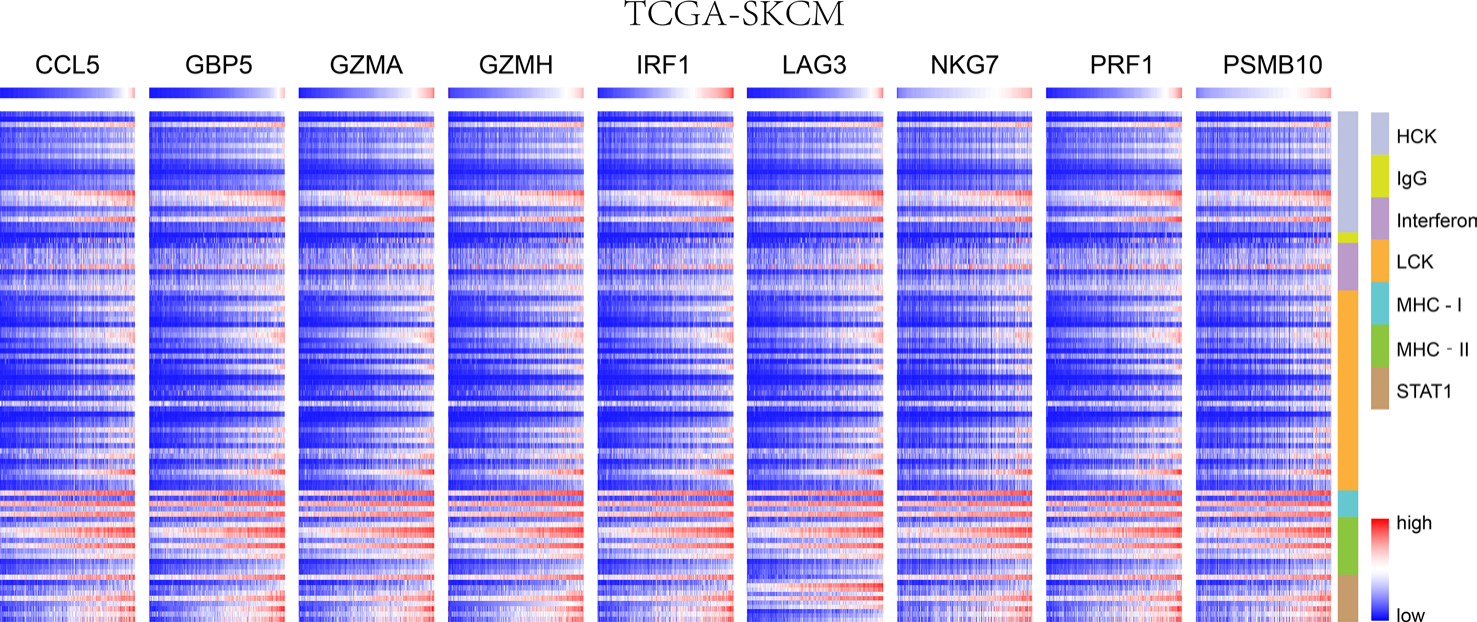
In SKCM-FPKM, CD8^+^ T cells infiltration promotes the correlation between genes and inflammatory and immune responses. These reactions were induced mainly by hematopoietic cell kinases, immunoglobulin G, interferon, lymphocyte-specific kinase, major histocompatibility complex class I, major histocompatibility complex class II, and activator of transcription 1.

**Figure 5:**
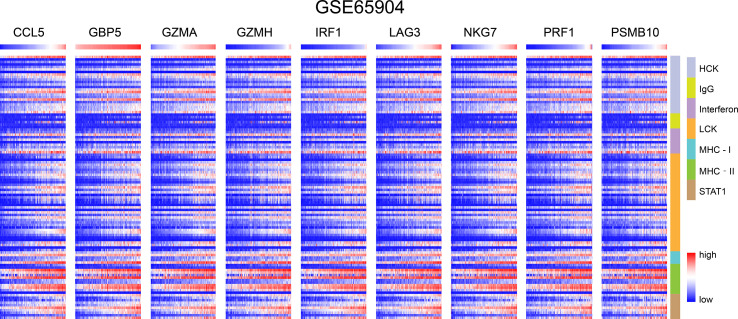
In GSE65904, CD8^+^ T cells infiltration promotes the correlation between genes and inflammatory and immune responses. These reactions were induced mainly by hematopoietic cell kinases, immunoglobulin G, interferon, lymphocyte-specific kinase, major histocompatibility complex class I, major histocompatibility complex class II, and activator of transcription 1.

We chose seven metagenes to analyze the correlation between the eight genes and immune responses, representing various inflammatory and immune responses. We found that CCL5, GBP5, GZMA, GZMH, IRF1, LAG3, NKG7, PRF1, and PSMB10 positively correlated with seven of these clusters in both TCGA-SKCM (**Figure 4**) and GSE65904 (**Figure 5**). The above results demonstrated that nine core genes were associated with stronger T cell response and immune response. The genes in the groups were uploaded in **Supplementary Table 4**.

### Survival Analysis and GSEA Analysis

**Figure 6:**
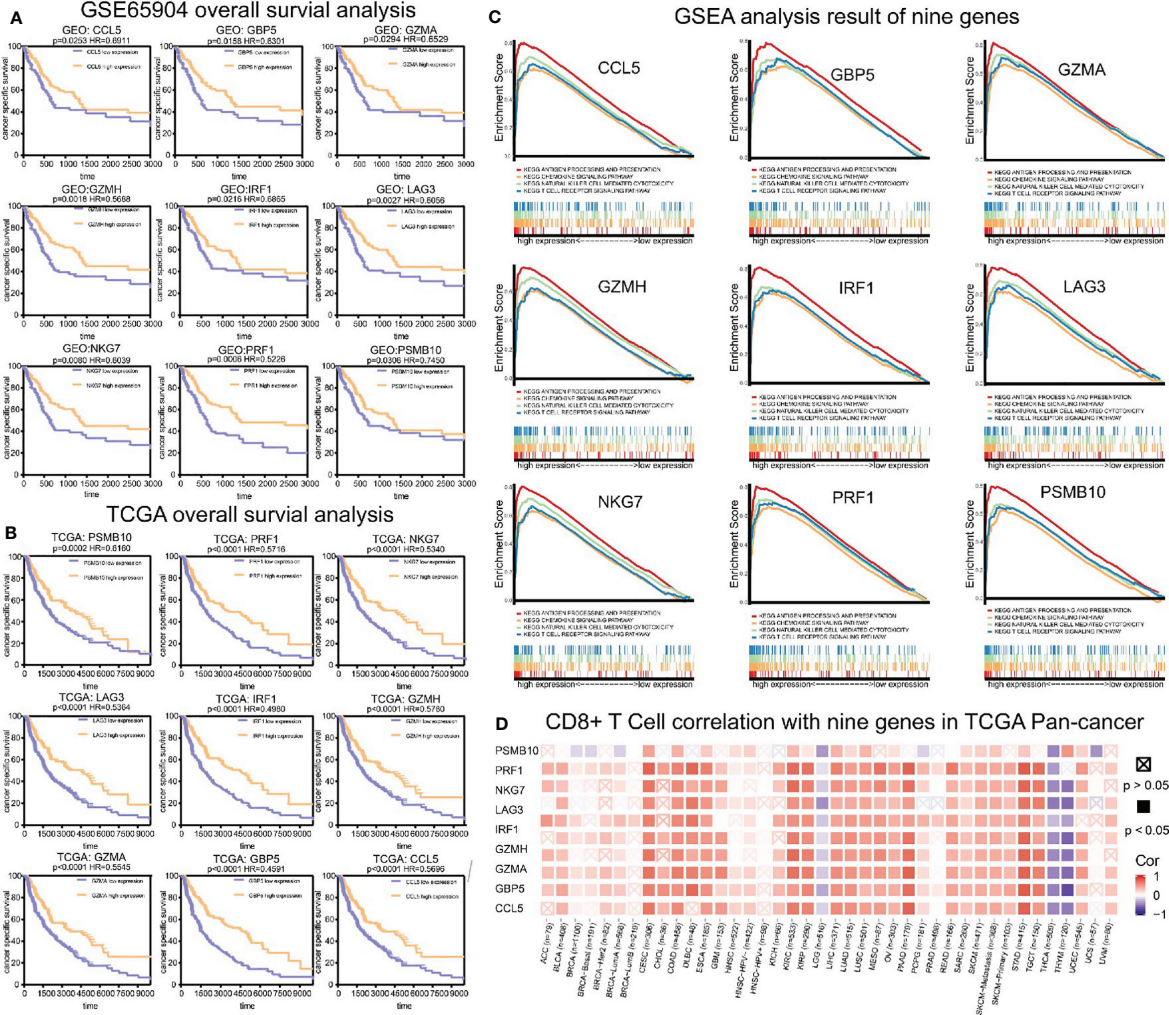
Survival analysis, GSEA analysis, and pan-cancer analysis in TIMER. **(A)** Survival analysis results of nine co-expressed genes promoting CD8^+^ T cells infiltration in GSE65904. The survival rate of the high expression group was significantly higher than that of the low expression group. **(B)** Survival analysis results of nine co-expressed genes promoting CD8^+^ T cells infiltration in SKCM-FPKM. The survival rate of the high expression group was significantly higher than that of the low expression group. **(C)** Results of GSEA analysis. These genes are mainly concentrated in the T cell receptor signaling pathway, antigen processing presentation, chemokine signaling pathway, and cytotoxicity mediated by natural killer cells. **(D)** The relationship between CCL5, GBP5, GZMA, GZMH, IRF1, LAG3, NKG7, PRF1, and PSMB10 and CD8^+^ T cells infiltration in other cancers.

To analyze their influence on cancer specific survival, we performed survival analysis. Patients in low expression groups for CCL5 (TCGA: *P* < 0.0001; GSE65904: *P* = 0.025), GBP5 (TCGA: *P* < 0.0001; GSE65904: *P* = 0.016), GZMA (TCGA: *P* < 0.0001; GSE65904: *P* = 0.029), GZMH (TCGA: *P* < 0.0001; GSE65904: *P* = 0.018), IRF1 (TCGA: *P* < 0.0001; GSE65904: *P* = 0.022), LAG3 (TCGA: *P* < 0.0001; GSE65904: *P* = 0.002), NKG7 (TCGA: *P* < 0.0001; GSE65904: *P* = 0.008), PRF1 (TCGA: *P* < 0.0001; GSE65904: *P* = 0.0006) and PSMB10 (TCGA: *P* < 0.0001; GSE65904: *P* = 0.0306) showed survival risks against high expression groups in TCGA-SKCM and GSE65904 (**Figures 6A, B**). GSEA analysis showed that the T cell receptor signaling pathway, antigen processing and presentation, chemokine signaling pathway, and nature killer cell-mediated cytotoxicity were related to the high expression group (**Figure 6C**). The P-values are displayed in **Table 2**. We found that these biological pathways were immune-related and were involved in tumor immunity.

**Table 2:** The results of GSEA analysis.

ID	Antigen processing and presentation		Chemokine signaling pathway		Nature killer cell mediated cytotoxicity		T cell receptor signaling pathway	
	NOM-p	FDR-q	NOM-p	FDR-q	NOM-p	FDR-q	NOM-p	FDR-q
CCL5	<0.0001	<0.0001	<0.0001	<0.0001	<0.0001	<0.0001	<0.0001	8.46E-04
GBP5	<0.0001	<0.0001	<0.0001	<0.0001	<0.0001	<0.0001	<0.0001	<0.0001
GZMA	<0.0001	<0.0001	<0.0001	<0.0001	<0.0001	<0.0001	0.0158	0.0150
GZMH	<0.0001	<0.0001	<0.0001	<0.0001	<0.0001	<0.0001	<0.0001	0.0004
IRF1	<0.0001	<0.0001	<0.0001	<0.0001	<0.0001	<0.0001	<0.0001	0.0005
LAG3	<0.0001	<0.0001	0.0110	0.0272	<0.0001	<0.0001	0.0020	0.0010
NKG7	<0.0001	<0.0001	0.0019	0.0143	<0.0001	<0.0001	0.0320	8.11E-05
PRF1	<0.0001	<0.0001	<0.0001	<0.0001	<0.0001	<0.0001	<0.0001	<0.0001
PSMB10	<0.0001	<0.0001	<0.0001	<0.0001	<0.0001	<0.0001	<0.0001	0.0002

### Timer Database Analysis

We demonstrated the role of CCL5, GBP5, GZMA, GZMH, IRF1, LAG3, NKG7, PRF1, and PSMB10 in melanoma. Next, we analyzed the correlation between these co-expression factors and CD8^+^ T cell infiltration in other types of cancers. CCL5, GBP5, GZMA, GZMH, IRF1, LAG3, NKG7, PRF1, and PSMB10 correlated to CD8^+^ T lymphocyte infiltration proportion in cutaneous melanoma, thyroid carcinoma, head and neck cell carcinoma, hepatocellular carcinoma, and lung adenocarcinoma squamous (**Figure 6D**).

### Single-Cell Analysis

**Figure 7:**
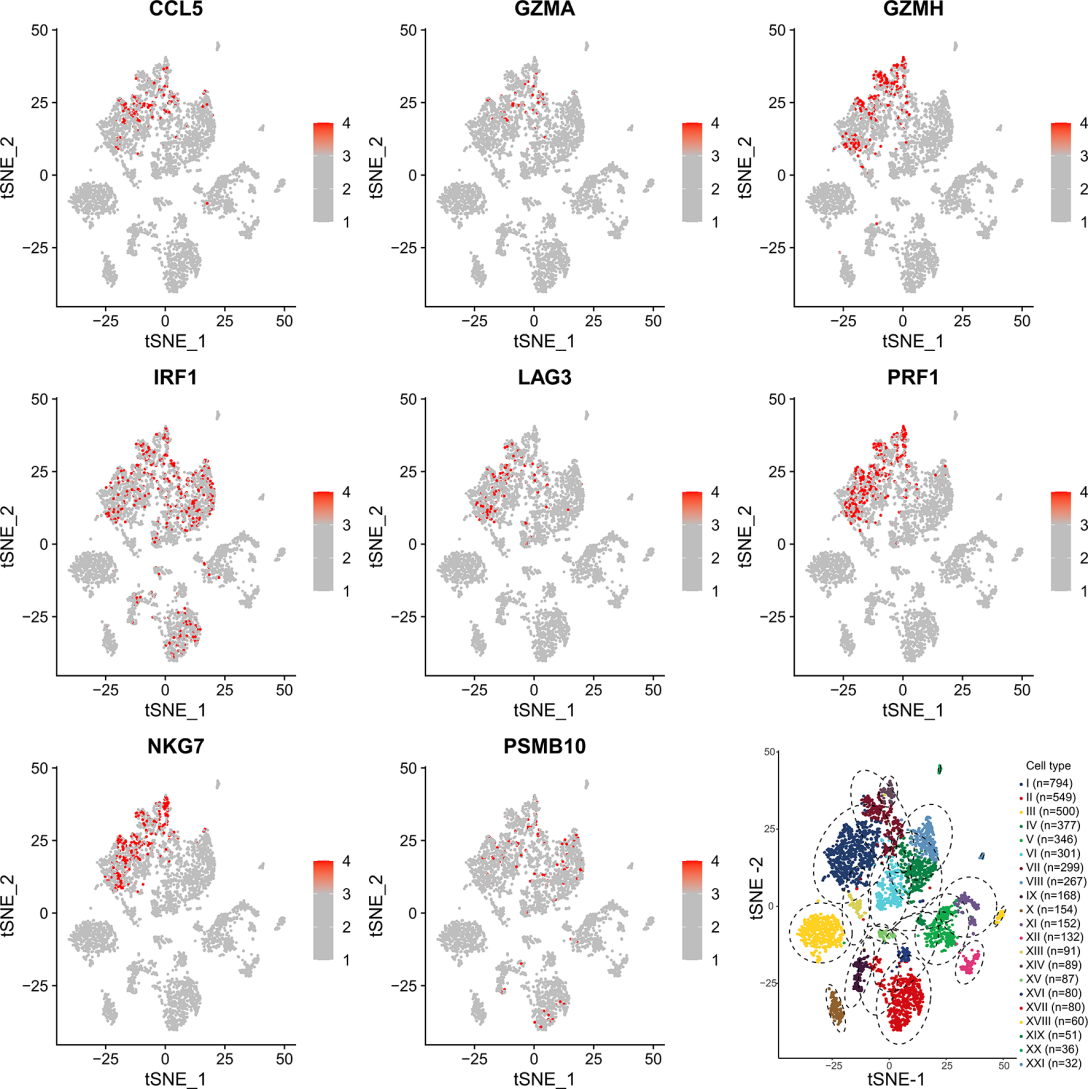
Single-cell sequencing verification of CCL5, GZMA, GZMH, IRF1, LAG3, NKG7, and PRF1 showing high expression in CD8^+^T cell subsets. I: CD8^+^ T cells. II: B cells. III: Melanocytes. IV: CD4^+^ T cells. V: Melanocytes. VI: CD4^+^ T cells. VII: CD8^+^ T cells. VIII: CD4^+^ T cells. IX: Macrophages. X: Melanocytes. XI: Melanocytes. XII: Melanocytes. XIII: CD8^+^ T cells. XIV: NK cells. XV: Fibroblasts. XVI: CD4^+^ T cells. XVII: B cells. XVIII: Endothelial cells. XIX: Melanocytes. XX: Melanocytes. XXI: Endothelial cells.

To determine which cells these genes came from, we conducted a single-cell analysis. After annotating the subsets with “SingleR”, we compared the results then found that the expression content of PSMB10, GZMA, GZMH, PRF1, CCL5 was relatively high in the CD8^+^T cell subsets, confirming our previous finding in TCGA-SKCM (**Figure 7**). The expression and distribution of CD8A, CD4, CD3E, and CD14 are shown in Supplementary **Figure 2**. The figure shows that these genes are more likely derived from CD8^+^T cells than other immune cells.

### 9-Gene Signature Cancer Prognosis Prediction

**Figure 8:**
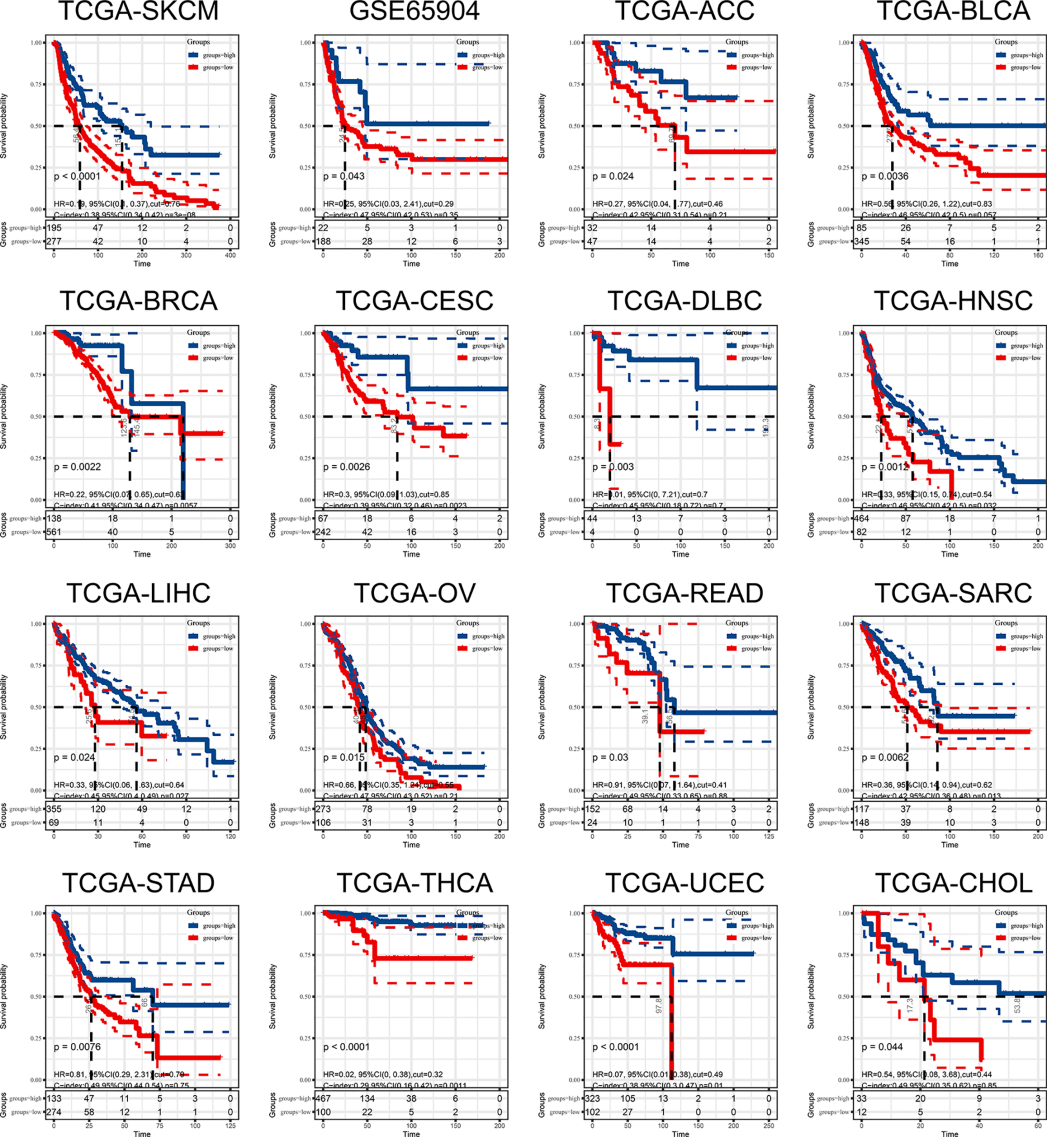
The ssGSEA score of 9-gene signature can be used as a prognostic protective factor in individual cancers.

In the above study, we identified a 9-gene signature correlated with CD8^+^T cells in melanoma. Based on the median grouping of 9-gene signature ssGSEA score, we figured out that patients with high expression of 9-gene signature in the TCGA and GSE65904 melanoma cohorts had a better prognosis. At the same time, we also demonstrated that patients in the group with high expression of 9-gene signature had better prognosis in multiple cancers of TCGA (**Figure 8**). The above results proves that patients with higher expression of the gene signature correlated with CD8^+^ T cell infiltration activated by IFN-γ have a better prognosis and survival.

**Figure 9:**
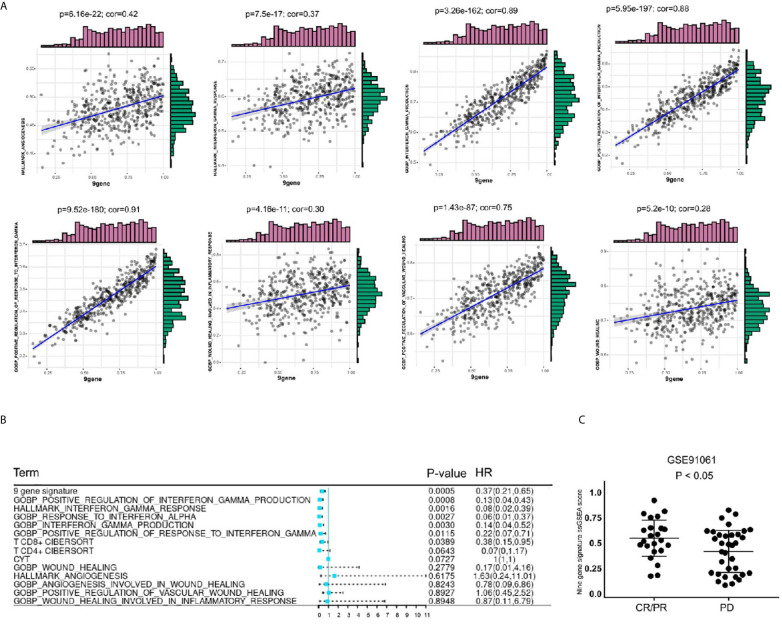
Comparison of gene sets with existing gene sets. **(A)** The 9-gene signature was positively correlated with angiogenesis, IFN-γ response, IFN-γ production, positive regulation of IFN-γ production, positive regulation of response to IFN-γ, wound healing, wound healing involved in inflammatory response, positive regulation of vascular wound healing. **(B)** In univariate COX regression analysis of disease-free survival of TCGA-SKCM, the gene-set of gene 9 had the most significant P value, p = 0.0005. **(C)** The level of 9 gene signature was higher in the immune checkpoint treatment response group of the GSE91061 cohort.

To further explore the value of this 9-gene signature, we analyzed its correlation with angiogenesis related pathways, wound healing related pathways, IFN-γ related pathways, etc. It turns out that the 9-gene signature was positively correlated with immune response (**Table 3**) angiogenesis, IFN-γ response, IFN-γ production, positive regulation of IFN-γ production, positive regulation of response to IFN-γ, wound healing, wound healing involved in inflammatory response, positive regulation of vascular wound healing, etc (**Figure 9A**). Later, through univariate analysis of 9-gene signature with CYTOLYTIC score, hallmark angiogenesis gene set, hallmark interferon gamma response gene set, CD4^+^T cell CIBERSORT score and CD8^+^T cell CIBERSORT score, we found that our 9-gene signature had more significant prognostic value (**Figure 9B**).

**Table 3:** The correlation between 9-gene signature with immune response related gene signature in TCGA-SKCM and GSE65904.

9-gene correlation	Interferon	LCK	MHC-I	MHC‐II	STAT1	IgG	HCK
GSE65904	0.632	0.886	0.787	0.835	0.903	0.578	0.794
TCGA-SKCM	0.594	0.917	0.616	0.874	0.881	0.625	0.843

### Validation of Immunotherapy Cohort

Based on these findings, we believe that the 9-gene signature is related to immunotherapy. We validated this in GSE78220, GSE91061 and GSE93157, which included immunotherapy follow-up data. In GSE91061 cohort, by comparing the 9-gene signature ssGSEA scores of the patients in the complete response (CR)/partial response (PR) group and the patients in the progressive disease (PD) group, it can be seen that the differences were statistically significant (**Figure 9C**). Then, we verified the survival predictive ability of 9-gene signature in GSE78220 and GSE93157 cohorts, and found that patients with low expression of 9-gene had a worse prognosis on immunocheckpoint inhibitor therapy (**Supplementary Figure 3**). This suggests that 9-gene signature may be one of the prognostic indicators for immune checkpoint therapy.

### Immunohistochemical Pathological Analysis

To investigate the clinical significance of PSMB10 in melanoma, immunohistochemical analysis of the protein expression levels of PSMB10 in and around carcinomas was performed in the melanoma cohorts of our hospital. One patient was a 58-year-old woman with lesions on the distal left middle finger, and one was a 72-year-old man with lesions on the right plantar skin. On gross observation, it was found that the color of the lesion skin was black and brown, with irregular shape with fuzzy boundary, and a few skin lesions were accompanied by necrosis, erosion, and ulcer.

**Figure 10:**
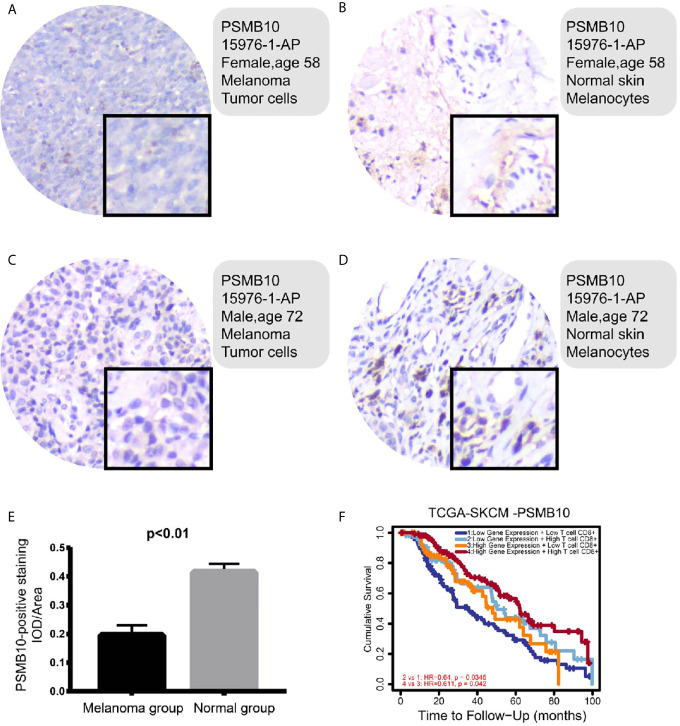
**(A–D)** In the immunohistochemical results, the PSMB10 expression level in normal tissue was higher than that of tumor tissue. **(E)** The IOD/area of paracancerous tissue was significantly higher than that of melanoma group. **(F)** In both groups with low PSMB10 expression and high PSMB10 expression, the group with low CD8^+^ T cell content had worse prognosis than the group with high CD8^+^ T cell content. In the low or high CD8^+^ T cell content groups, the prognosis of the low PSMB10 expression group was worse than that of the high PSMB10 expression group.

Immunohistochemical detection showed that PSMB10 was positive in the two malignant melanoma specimens, a positive rate of 100%. PSMB10 had lower staining intensity in cancer tissue, while PSMB10 had higher staining intensity in normal tissue (**Figures 10A–D** ). At the same time, we supplemented 15 quantitative immunohistochemical analyses of melanoma and paracancerous tissues, and the results showed that the IOD/Area of PSMB10 in paracancerous samples was higher (**Figure 10E**). We then combined the PSMB10 gene expression and CD8^+^T lymphocytes to draw the survival curves of multiple groups. Patients with high levels of CD8^+^T lymphocytes and PSMB10 had the best prognosis, while patients with low levels of CD8^+^T lymphocytes and PSMB10 had the worst prognosis (**Figure 10F**).

## Discussion

In this research, we selected samples from two data sets, calculated CD8^+^ T cell infiltration in melanoma tissue samples using the CIBERSORT package, identified co-expressed genes related to CD8^+^ T cell infiltration using the WGCNA algorithm, and performed preliminary screening based on intersection results. We subsequently selected nine genes that the value of module correlation rank the top in the WGCNA analysis. We performed functional enrichment in a biological process in which these genes were enriched in the IFN-γ mediated signaling pathway and the antigen presentation and processing pathway. The obtained 9-gene signature was confirmed to be related to the activation of IFN-γ in the correlation analysis of the immune response related gene set and IFN-γ related gene set.

In GSEA analysis, the pathways enriched in the high expression group of critical genes were associated with tumor immunity. Essential genes play essential roles in CD8^+^ T cells infiltration in some cancers: CCL5, GBP5, GZMA, GZMH, IRF1, LAG3, NKG7, PRF1, and PSMB10 were identified as promoting factors for CD8^+^ T cell infiltration, all of which had independent prognostic effects.

Harlin et al. found that CCL5 produced by melanoma cells helped chemokine recruitment of CD8^+^ effector T cells (26). PRF1 is expressed by a protein called perforin, expressed by CD8^+^ T cells, and is essential for cell-mediated cytotoxicity and effective control of pathogens (27). Taube et al. demonstrated that CD8A, PRF1, and CCL5 were overexpressed in PD-L1^+^ melanoma and were involved in the activation of CD8^+^ T cells (28). GZMA is the most abundant protease in cytotoxic particles of NK cells (29). Inoue et al. examined mRNA levels of immune-related genes in melanoma patients before and after immunotherapy with nivolumab and found increased CD8 and GZMA expression levels (30). IRF1 is an effective antiviral, antitumor, and immunoregulatory protein. CD8^+^ T cells in IRF1-deficient melanoma showed increased cytotoxicity, the expression of PD-L1 was upregulated, and tumor growth was more easily restored (31).

LAG3 is the third alternative inhibitory receptor targeted in the tumor microenvironment (32). Frohlich et al. found that LAG3 methylation in melanoma tissues was associated with CD8^+^ T cells infiltration and IFN-γ signaling (33). NKG7 is one of the most highly expressed genes in NK cells and is essential for cytotoxic degranulation of NK cells and CD8^+^ T cells and the activation and pro-inflammatory response CD4^+^ T cells (34). Fairfax et al. found that patients who responded to immunotherapy had more CD8^+^ T cell-related large clones overexpressed gene NKG7 than did non-responders (35). GBP5 and PSMB10 have been reported in tumors as part of the downstream interferon genes and are also considered to be the coordinators of tumor disease immunity (36, 37). Li et al. reported that GZMH is a T cell gene in single-cell analysis of T cells in melanoma (38).

Nine co-expressed genes correlated with CD8^+^ T cell infiltration were significantly enriched in the IFN-γ pathway, suggesting that IFN-γ may be closely related to CD8^+^ T cell infiltration. Dangaj et al. found that T cell infiltration required tumor cell-derived CCL5, and CXCL9 secretion by IFN-γ differentiated myeloid cells was amplified; in immunoreactive and immune-responsive tumors, the synergistic effect of tumor-derived CCL5 and IFN-γ-induced CXCR3 ligand secreted by bone marrow cells is the key to coordinate T cell infiltration (39). Jia et al. analyzed the distribution of tumor-infiltrating T cells and the expression of PD-L1 in the orthotopic murine glioma model; GBP5, IRF1, as IFN-γ-induced genes were positively correlated with PD-L1 scores as a measure of alternative IFN-γ levels (40). It was proposed that tumors with high neutrophil burdens are characterized by inadequate T cell responses, with decreased cytotoxic T cell genes such as CD8A, CD8B, GZMA, and GZMB. There was decreased infiltration of CD3^+^ T cells and CD8^+^ T cells and decreased expression of IFN-γ-related genes (41). Lichtenegger et al. found that blocking LAG-3 led to higher T cell activation and an increase in IFN-γ secretion compared with inhibition of other pathways; they concluded that the novel immune response was strongly enhanced by blocking LAG-3 or blocking both LAG3 and PD-1 (42). The promoter region of the PSMB10 gene contains two IFN stimulus-response elements cross-regulated by IFN; this was confirmed by in vitro mutagenesis (43).

Curtsinger et al. stimulated CD8^+^ T cells in mice using antigen-specific B7-1. They found that they could rapidly produce a small amount of IFN-γ, with the production peaking at about 8 hours and decreasing after 24 hours (44). When CD8^+^ T cells are exposed to mild temperatures, they promoted the production of specific IFN-γ, which increased the lethality of tumor target cells (45). Karachaliou et al. treated 21 melanoma patients with pembrolizumab and found that patients with high IFN-γ expression had significantly longer progress-free survival than those with low IFN-γ expression (46).

In summary, CCL5, GBP5, GZMA, GZMH, IRF1, LAG3, NKG7, PRF1, and PSMB10 are co-expression genes related to CD8^+^ T cell infiltration. The lack of CD8^+^ T cells in central tumor areas has become a significant obstacle to immunotherapy for solid tumors, especially melanoma. Therefore, novel therapeutic strategies that promote the accumulation of CD8^+^ T cells in central tumor regions are urgently needed.

## Data Availability Statement

Publicly available datasets were analyzed in this study. The data set of this article was downloaded from the open source database TCGA and GEO. TCGA: (http://cancergenome.nih.gov/); GEO: GSE65904, GSE78220, GSE72056, GSE91061 and GSE93157 were downloaded from the GEO database (http://www.ncbi.nlm.nih.gov/geo/).

## Ethics Statement

The studies involving human participants were reviewed and approved by Medical Ethics Committee of Fuzhou No. 1 Hospital Affiliated with Fujian Medical University. The patients/participants provided their written informed consent to participate in this study.

## Author Contributions

KY and YW conceived ideas, designed methods, analyzed and visualized the data, and wrote the draft paper. YL contributed analysis tools and analyzed the data. ZY managed the project, supervised and led the experiment, and reviewed and revised the paper. All authors contributed to the article and approved the submitted version.

## Conflict of Interest

The authors declare that the research was conducted in the absence of any commercial or financial relationships that could be construed as a potential conflict of interest.

## Supplementary Material

Supplementary Figure 1 Analysis of the microenvironment of immune infiltration. **(A)** Infiltration ratio of 22 immune cells in SKCM-FPKM. **(B)** Tumor purity, estimated score, immune score, and a stromal score of SKCM-FPKM were calculated by immune microenvironment analysis as phenotypic information for WGCNA analysis. **(C)** Infiltration ratio of 22 immune cells in GSE65904. **(D)** Tumor purity, estimated score, immune score, and a stromal score of GSE65904 were calculated by immune microenvironment analysis as phenotypic information for WGCNA analysis.

Supplementary Figure 2 The expression of cell markers CD3E, CD4, CD8A and CD14 in single cell analysis.

Supplementary Figure 3 The clinical response of immunotherapy was better in the 9-gene ssGSEA low-rated group **(A)** GSE78220 **(B)** GSE93157.
