# Supplementary figures and images for: 9-Gene Signature Correlated With CD8+ T Cell Infiltration Activated by IFN-γ: A Biomarker of Immune Checkpoint Therapy Response in Melanoma
Source: Front Immunol. 2021 Jun 17;12:622563. doi: 10.3389/fimmu.2021.622563 (PMC8248551; doi:10.3389/fimmu.2021.622563)

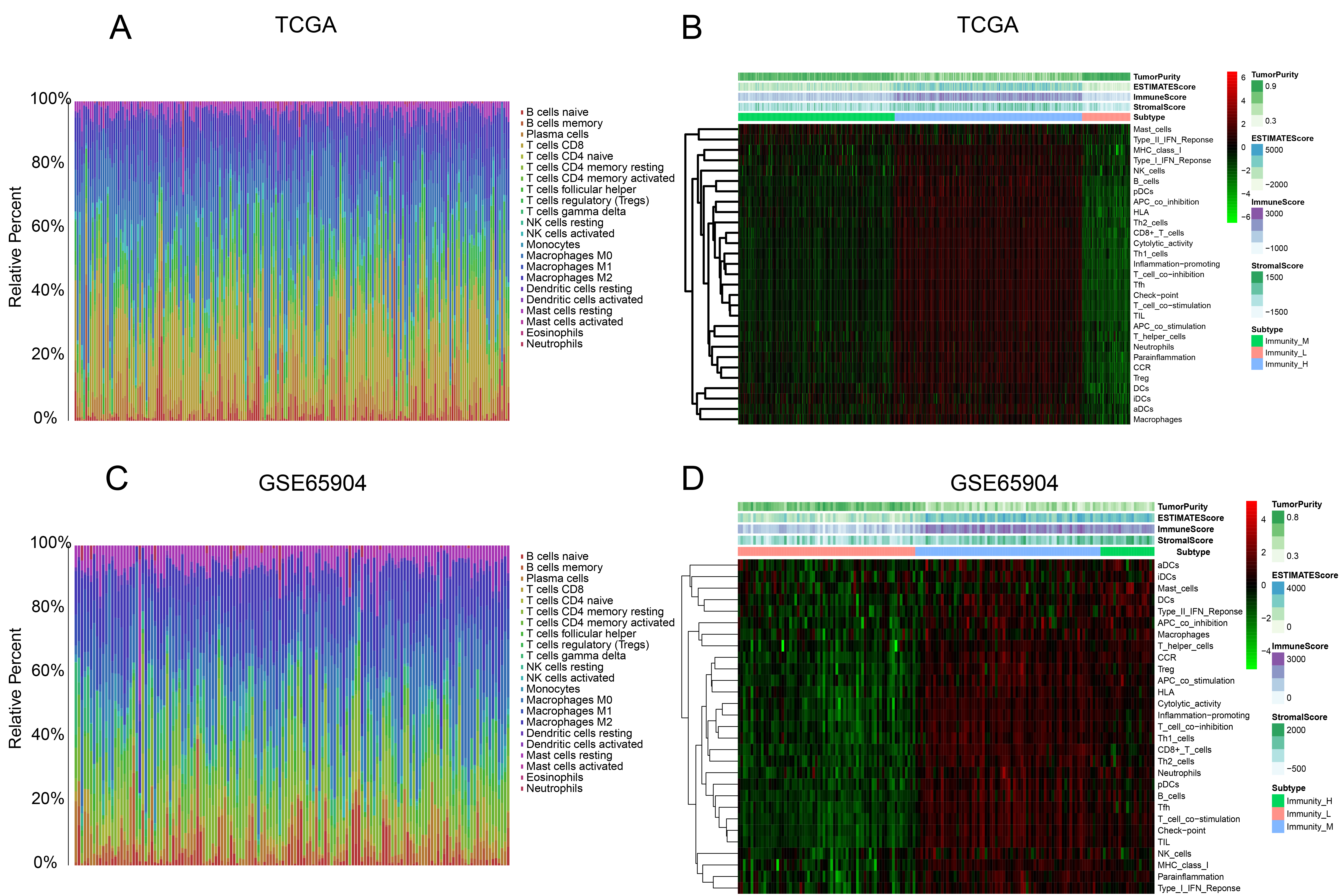

Supplement: Supplementary Figure 1 — Analysis of the microenvironment of immune infiltration. (A) Infiltration ratio of 22 immune cells in SKCM-FPKM. (B) Tumor purity, estimated score, immune score, and a stromal score of SKCM-FPKM were calculated by immune microenvironment analysis as phenotypic information for WGCNA analysis. (C) Infiltration ratio of 22 immune cells in GSE65904. (D) Tumor purity, estimated score, immune score, and a stromal score of GSE65904 were calculated by immune microenvironment analysis as phenotypic information for WGCNA analysis. [file Image_1.tif]

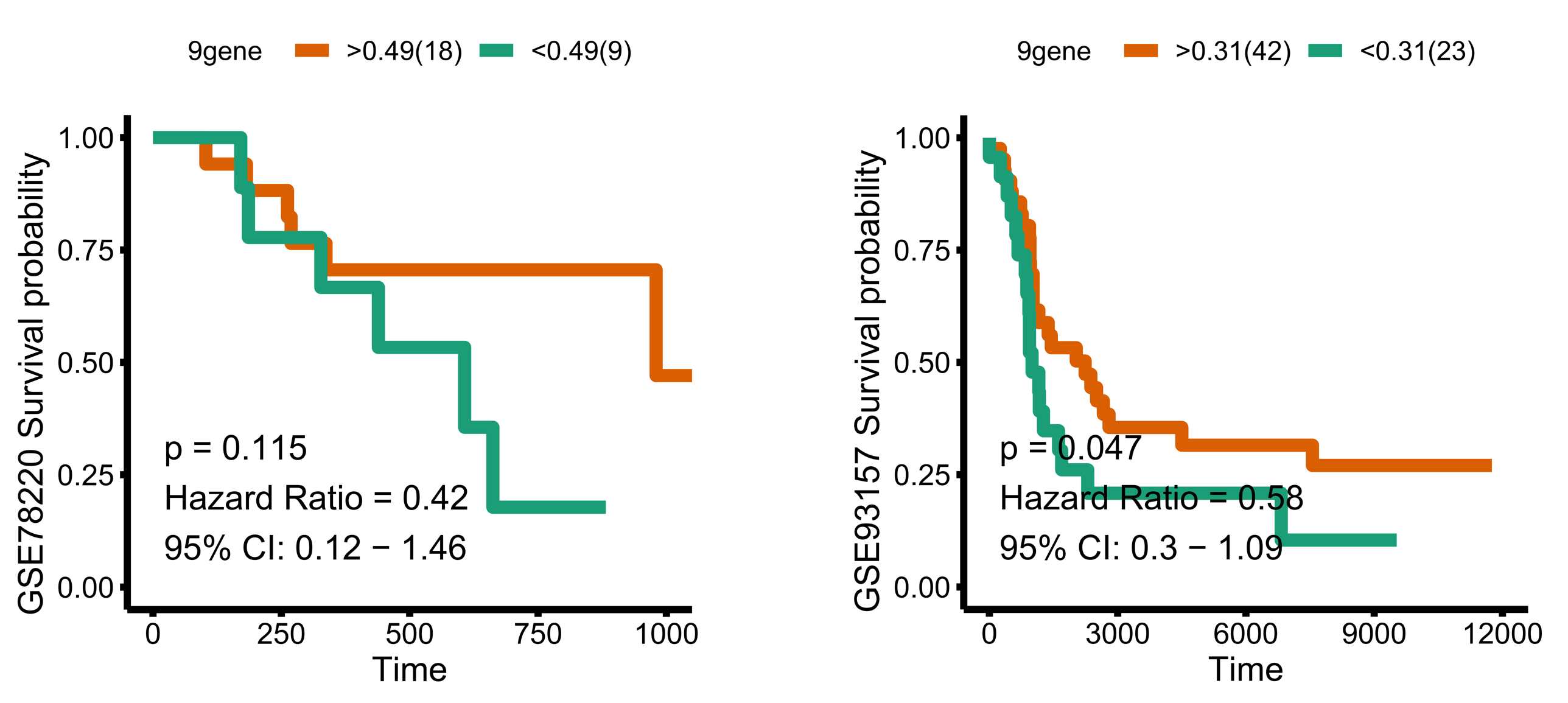

Supplement: Supplementary Figure 3 — The clinical response of immunotherapy was better in the 9-gene ssGSEA low-rated group (A) GSE78220 (B) GSE93157. [file Image_3.tif]
