# Supplementary material for: 9-Gene Signature Correlated With CD8+ T Cell Infiltration Activated by IFN-γ: A Biomarker of Immune Checkpoint Therapy Response in Melanoma
Source: Front Immunol. 2021 Jun 17;12:622563. doi: 10.3389/fimmu.2021.622563 (PMC8248551; doi:10.3389/fimmu.2021.622563)
Supplement: Supplementary file 5 [file Table_2.docx]

| Supplementary Table 2. The top 20 Gene significance for CD8+ T Cells related genes in TCGA | | |
| --- | --- | --- |
| ID | GS.T.cells.CD8+ | P-Value |
| CCL4 | 0.736091387 | 3.22E-32 |
| CD8A | 0.722091291 | 1.98E-29 |
| CCL5 | 0.715026003 | 6.32E-28 |
| PDCD1 | 0.714267321 | 5.48E-28 |
| CD8B | 0.71398938 | 4.22E-22 |
| IRF1 | 0.703799641 | 1.56E-21 |
| LAG3 | 0.693420616 | 6.79E-19 |
| PRF1 | 0.690723744 | 6.55E-19 |
| UBE2L6 | 0.690100702 | 8.78E-18 |
| PSMB10 | 0.689228002 | 5.39E-18 |
| IL2RB | 0.687954368 | 9.69E-16 |
| CCL4L2 | 0.678041891 | 1.73E-10 |
| TAP1 | 0.676344852 | 1.38E-10 |
| NKG7 | 0.67401899 | 1.30E-10 |
| PSMB9 | 0.66998417 | 1.27E-10 |
| GZMA | 0.667739065 | 1.11E-10 |
| GZMH | 0.663406101 | 1.03E-10 |
| GCH1 | 0.649634557 | 1.01E-10 |
| PSME2 | 0.648029006 | 5.66E-09 |
| CALHM6 | 0.64484191 | 9.85E-09 |
| GS: Gene significance | | |
