# Supplementary material for: 9-Gene Signature Correlated With CD8+ T Cell Infiltration Activated by IFN-γ: A Biomarker of Immune Checkpoint Therapy Response in Melanoma
Source: Front Immunol. 2021 Jun 17;12:622563. doi: 10.3389/fimmu.2021.622563 (PMC8248551; doi:10.3389/fimmu.2021.622563)
Supplement: Supplementary file 6 [file Table_3.docx]

| Supplementary Table 3. The top 20 Gene significance for CD8+ T Cells related genes in GSE65904 | | |
| --- | --- | --- |
| ID | GS.T.cells.CD8+ | P-Value |
| CCL5 | 0.705645256 | 1.85E-18 |
| NKG7 | 0.683734095 | 5.15E-17 |
| GZMH | 0.657209852 | 2.00E-15 |
| GZMK | 0.637643079 | 2.37E-14 |
| GZMA | 0.636954799 | 2.57E-14 |
| IRF1 | 0.611594302 | 4.90E-13 |
| EOMES | 0.591550333 | 4.20E-12 |
| IFNG | 0.577640184 | 1.71E-11 |
| PSMB10 | 0.564490778 | 6.10E-11 |
| LAG3 | 0.558528155 | 1.07E-10 |
| GBP5 | 0.555700451 | 1.38E-10 |
| PRF1 | 0.537382717 | 7.07E-10 |
| CST7 | 0.523473172 | 2.29E-09 |
| HLA-DQA1 | 0.521555402 | 2.68E-09 |
| CD2 | 0.507100944 | 8.54E-09 |
| SH2D1A | 0.501762766 | 1.29E-08 |
| HLA-DPA1 | 0.499733912 | 1.51E-08 |
| PYHIN1 | 0.496612555 | 1.91E-08 |
| GBP1 | 0.486056029 | 4.20E-08 |
| IL2RB | 0.482885609 | 5.29E-08 |
| GS: Gene significance | | |
